# Comparative Studies of Perianal Structures in Myrmecophilous Aphids (Hemiptera, Aphididae)

**DOI:** 10.3390/insects13121160

**Published:** 2022-12-16

**Authors:** Natalia Kaszyca-Taszakowska, Mariusz Kanturski, Łukasz Depa

**Affiliations:** Institute of Biology, Biotechnology and Environmental Protection, Faculty of Natural Sciences, University of Silesia in Katowice, Bankowa 9, 40-007 Katowice, Poland

**Keywords:** ultramorphology, scanning electron microscopy, ants, mutualism, lotus effect

## Abstract

**Simple Summary:**

Aphids involved in obligatory mutualistic relationship with ants are believed to have short cauda and elongated, vertical anal plate to allow keeping the honeydew droplet until removed by ants. Our study indicated no such correlation of morphology and ant-attendance between facultatively and obligatory myrmecophilous aphids. Review through microstructure of perianal cuticle indicated that the microrelief of cuticle may play a role in keeping excreted honeydew away from aphid body until removed by ant worker.

**Abstract:**

There are three types of relationships between aphids and ants: non-myrmecophilous, obligatory and facultatively myrmecophilous. The degree of involvement in this mutualism is believed to be corelated with morphological adaptations of perianal structures. In this manuscript, we analyzed the differences of these structures in obligatorily (18 species) and facultatively (10 species) myrmecophilous aphids. Scanning electron microscopy (SEM) and light stereoscopic microscopy (LSM) techniques were used for these studies. Comparison of structures showed no strict relationship between their morphology and the degree myrmecophily, with certain indication that the microsculpture of perianal cuticle may play role in protection of aphids against honeydew droplet in facultatively myrmecophilous aphids.

## 1. Introduction

Aphids and ants are two abundant and highly successful insect groups, which in the zone of temperate climates often live in the same habitat and therefore are likely to interact with one another [1]. Mutualistic associations between them can be viewed like any other mutualistic interaction: reciprocal exploitations that provide net benefits for both partners [2,3,4]. There are many not mutually exclusive hypotheses accounting for ant attendance and most of them are framed within a cost-benefit perspective [5]. For aphids, ant attendance usually entails various benefits, including better protection against predators and parasitoids [6,7,8,9,10], reduced risk of contracting fungal infections due to the grooming action of the ants with the removal of exuviae and infected individuals [6,11,12], transfer to more suitable locations [13] and reduced indirect competition with untended aphids [14]. The ants, in turn, benefit nutritionally from harvesting honeydew, as it is rich in mono-, di- and trisaccharides and amino acids [15,16,17,18,19,20,21,22]. In some cases, ant attendance, however, may also have some costs, given that the production of an unusually large quantity or high quality of honeydew is likely to be energetically expensive [1,9,23]. The balance of these costs and benefits probably explains why only a third of the aphid species in Europe is considered to be obligate mutualists, and another third is only facultative mutualists, with the remainder not being ant-tended [24,25,26].

This unique relationship called trophobiosis occurs in many aphids to a very different extent. It is usually divided into two types: the obligatory and the facultative one, although this division seems to be a simplification, not fully representing a diversity of mutual relationships [27]. The first one comprises aphid species which always occur with ants, and these aphids probably cannot live without ant attendance (e.g., *Stomaphis quercus*) [28]. The second type involves aphids which may or may not be attended by ants. The reasons for these divergent developments are still largely unknown. Among hemipteran species, which feed on the same host plant and even on the same plant organ, some may be always attended, while others are never visited by ants [29].

**Figure 1:**
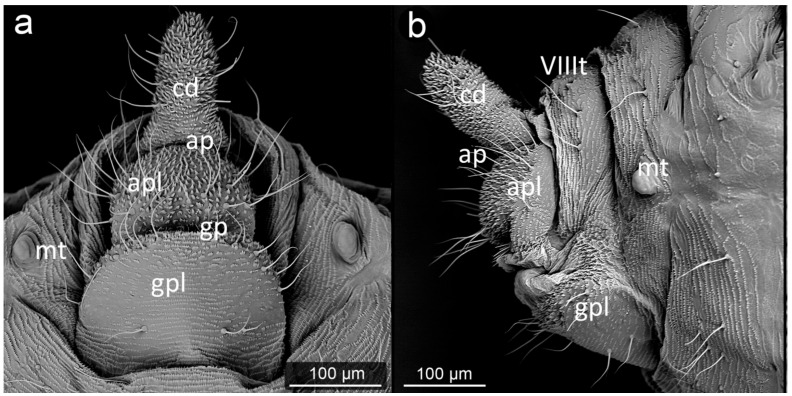
Scanning electron micrographs (SEM) of *Aphis fabae* showing the general morphology of aphid abdominal and perianal structures: (**a**)—ventral view, (**b**)—lateral view (t—tergite, cd—cauda, apl—anal plate, gpl—genital plate, mt—marginal tubercle, ap—anal pore, gp—genital pore, VIIIt—abdominal tergite VIII).

The perianal structures in aphid morphology are the main area of direct contact between aphids and ants visiting them. Although they seem to play an important role in transferring honeydew droplet from the anal pore of aphid to ant mouthparts [30], their modifications are regarded just an adaptation to mutualism with ants. The perianal structures in aphids include the posterior abdominal elements: tergites, sternites and structures resulting from their modification (Figure 1). They include (from the dorsal side) tergite VIII (VIIIt)—usually smaller than the other seven tergites, but with the classic structure of a half-rim, constituting a support for further perianal structures; cauda (cd)—transformed tergite X, with high morphological variability between species, from short and tightly adjacent to the body to highly elongated, club-shaped outgrowth. It is always strongly sclerotized due to its function of holding the drops; underneath it, in the cavity, there is anal pore (ap). Then, moving onto the ventral structures, anal plate (apl)—transformed sternite X, usually taking the form of a more or less rectangular plate and sternite VIII constituting the genital plate (gpl)—visible usually in adults, highly sclerotized, with genital pore (gp) between them. Sternite IX in aphids is extremely reduced, existing only in form of gonapophyses, which are remnants of reduced ovipositor, as parthenogenetic viviparity is dominant way of reproduction in aphids.

For most aphid species involved in obligate trophobiosis, the main morphological adaptation serving to facilitate transfer of the droplet of honeydew to ants is shortened cauda and longer setae in the anal region. These setae are widely believed to form a sort of basket that holds honeydew droplet until it is taken by ants [27,30,31]. These sets of features were discovered and described in 1907 by Mordvilko [32,33,34,35], who called them “trophobiotic organ”. Since then, practically no extensive research has been conducted on the function and construction of the trophobiotic organ apart from the revision of Zwölfer’s data [32,33,34] on Anoeciinae, subterranean Lachninae and Eriosomatinae *s*. *lat*. Made by Kanturski et al. [31], later extended also on representatives of the genus *Stomaphis* (Lachninae) [36]. These studies, however, similarly to works by Zwölfer, referred only to aphid species living either underground on roots of plants or conducting cryptic life mode on tree trunks (e.g., *Stomaphis* spp.).

The aim of this study is to characterize the perianal structures of several obligatory and facultatively myrmecophilous aphid species living in more open spaces, mainly on the overground parts of plants, in search for potential differences in these structures which might be connected to trophobiosis. The research focused on qualitative analysis, namely the character of cuticular microsculpture of perianal region, and quantitative, in the form of size relations between cauda and anal plate.

## 2. Material and Methods

### 2.1. Taxon Sampling

All aphid species were collected from September 2017 to October 2019 in Upper Silesia region of Poland. Each species was collected into a set of tubes containing 70% ethanol (EtOH) (Odczynniki Sp. z o.o. Sp. k., Katowice, Poland) for slide preparation, 2.5% glutaraldehyde (GA) (Odczynniki Sp. z o.o. Sp. k., Katowice, Poland) for scanning microscope (Phenom XL, Phenom-World B.V., Eindhoven, The Netherlands) and sometimes 100% ethanol for molecular identification.

The list of species studied in this research is presented in Table 1. It was very difficult to assign aphid species to category of obligate myrmecophiles unequivocally, as it seems that such division is tentative simplification due to lack of credible, large-scale research and variability of this relationship [27]. Therefore, the applied division to obligatory and facultative myrmecophiles was provisional, based on summarized data presented in Heie [37,38,39,40,41,42], Blackman and Eastop [43] and Depa and Wojciechowski [44] on the frequency of ant-attendance of particular aphid species. Only in cases where data in sources (if provided) concurred that the species was ant-attended we treated it as obligate myrmecophile. If there was any disagreement, we treated such species as facultative myrmecophile. The species selected for this study represented various life strategies, living both on stems and shoots of herbaceous plants as well as leaves, shoots and branches of trees, both deciduous and coniferous. For some of them, it was difficult to unequivocally assign them to facultatively myrmecophilous representatives of subfamily (e.g., Calaphidinae, Chaitophorinae, Thelaxinae and Lachninae). Comparisons of these species are set to concern also non-myrmecophilous species (Kaszyca-Taszakowska *in prep*.). The highest number of samples represented the subfamily Aphidinae, which comprises aphids with very diverse host plant associations, life strategies, morphology and relationships with ants.

### 2.2. Material

After mounting on the microscopic slide with the method described by Kanturski and Wieczorek [45] the specimens were identified using keys by Blackman and Eastop [43], Wojciechowski [46], Wojciechowski et al. [47] and Heie [37,38,39,40,41,42]. Systematic order was applied according to Favret [48]. The material (mounted specimens) was deposited in the entomological collection CEBD HEMIPTERON (DZUS), University of Silesia in Katowice. Adult female aphids, both apterae and alatae, were used for this study.

Samples of the genus *Dysaphis* were determined using key by Blackman and Eastop [43] and their identification was confirmed with molecular barcoding. The DNA isolation and PCR reaction with application of mitochondrial marker COI followed methods described by Mróz et al. [49]. The sequences were compared with barcode sequences deposited in Genbank.

### 2.3. Scanning Electron Microscopy

Scanning electron microscopy was used as the principal method in this study. Depending on the availability of the collected aphids, from two to ten individuals have been fixed and analyzed using SEM. The specimens for SEM studies were prepared using method by Kanturski et al. [50], but it was modified (phosphotungstic acid stage was omitted) as follows.

The samples from glutaraldehyde were prepared following standard protocol: maceration in 96% ethanol (approximately 10 min), and two rounds of maceration in absolute ethanol for 15 min. After this, samples were put into plastic tubs, and chloroform was added for 24 h. In the next step, we used hexamethyldisilazane (HMDS) solution with EtOH in the proportion of 1/3 for 20 min, 1/2 for 30 min, 2/3 for 45 min, 3/3 for 30 min (2 × 15min). They were glued to 32 mm diameter tables with carbon discs in different positions, allowing a detailed analysis of the species from different perspectives. Exceptions were species with a small number of preserved specimens, in which case we tried to make the picture of the aphid entire anal structure as full as possible. SEM micrographs were obtained using Phenom XL field emission scanning electron microscope (Phenom-World B.V., Eindhoven, The Netherlands) at 5, 10 and 15 kV accelerating voltage with a BackScatter Detector (BSD). The figures were prepared using Adobe Photoshop CS6 (Adobe Inc., San Jose, CA, USA) graphic editor.

### 2.4. Light Microscopy

Light microscopy was used to identify species and as a complementary method of documentation. In cases where it was impossible to obtain images of acceptable quality in SEM, mostly due to badly fixed specimens, figures were implemented with images from light microscopy (LSM). The focus-stacked photographs were obtained using Leica DM 3000 upright light microscope with Leica MC 190 HD digital camera and Leica Application Suite 4.12.0 software (Leica Camera AG, Wetzlar, Germany). The figures were prepared using Adobe Photoshop CS6 graphic editor.

### 2.5. Morphological Measurements

A characteristic feature developed from the tergite of abdominal segment X. The sternite of this segment transformed into a sclerotized structure called the anal plate. The tergite of abdominal segment IX atrophied, and the sternite reduced to the rudimentary gonapophyses. Abdominal tergites VII and VIII formed the subgenital plate [31]. The abdomen of Aphididae originally consisted of ten segments, of which only nine remained and were visible [51]. Abdominal segments VII, VIII, cauda, anal plate, subgenital plate and rudimentary gonapophyses are considered to be the end of abdomen [37,52]. The anal pore of Aphididae is located on the border between cauda and anal plate, whereas the genital aperture is located between the genital plate and rudimentary gonapophyses which lie directly under the anal plate.

**Figure 2:**
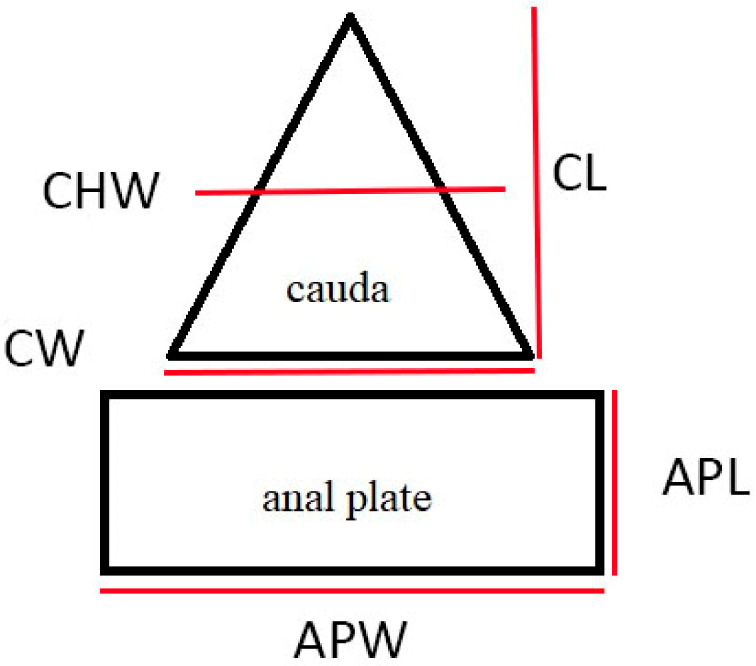
Scheme of measurements of perianal structures applied in this study: CHW—cauda half width; CL—cauda length; CW—cauda width; APW—anal plate width; APL—anal plate length.

The following qualitative characters were taken into account during the descriptions: the shape of anal plate, shape and sclerotization of ABD VIII (abdominal tergite VIII), arrangement of setae and their shape on ABD VIII, cauda and anal plate. The descriptions were implemented by measurements of distinctive morphological features, including anal plate length, anal plate width in the widest place, cauda length, cauda width at the base and cauda half width (Figure 2). Perianal structure measurements were measured from microscopic slides and partly from SEM with a Leica DM 3000 upright light microscope with Leica MC 190 HD digital camera and Leica Application Suite 4.12.0 software. For statistical analysis of difference between the ratios, standard t-test was performed for unpaired samples, with *p* < 0.05.

It must be noted here that measurements given in descriptions of perianal structures based on SEM may not reflect morphometrics typical of species based on studies of microscopic slides. This is due to the fact that during mounting for microscopic slides specimen is pressed thin, so the structures are somewhat deformed. Additionally, also during mounting for SEM, these structures may become deformed during drying and so not always the measurements are presented, only in specimens where it was possible to measure the structure with significant confidence. However, the applied method did not disturb the chitinous microsculpture of aphid cuticle, so wherever it remained exposed to the microscopic observation, it kept its original structure.

## 3. Results

The results of measurements of perianal structures as well as ratios between them are presented in Table 1, and in details in Tables S1–S3. The difference in length to width ratio of anal plate between obligatorily and facultatively myrmecophilous Aphidinae was statistically significant, while between all studied species it was not significant. It seems that in obligatorily myrmecophilous aphids the anal plate is shaped close to square (apl/apw = 1.07) while in facultatively myrmecophilous aphids it was much longer (or higher) than it is wide (apl/apw = 1.81). In addition, in case of cauda length to width ratio, in both groups, we noticed statistically significant difference between obligatorily and facultatively myrmecophilous species, with cauda significantly longer than it is wide in obligatorily myrmecophilous species (cl/cw ratios 1.08 to 1.49 in all species and 1.17 to 1.75 in Aphidinae) (Table 1).

The detailed descriptions of perianal structures of studied species are provided. In following sections.

### 3.1. Obligatorily Myrmecophilous Aphids

**Figure 3:**
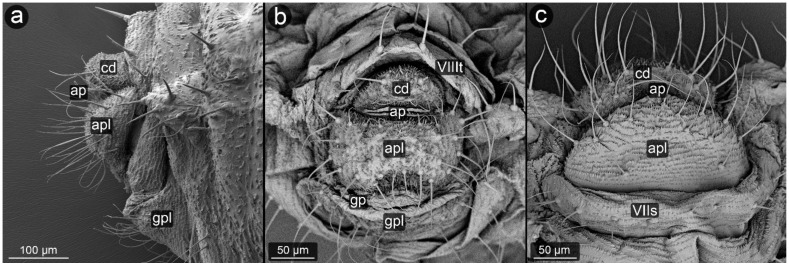
Perianal structure of *Glyphina betulae* in SEM; (**a**)—lateral view, (**b**)—rear view, (**c**)—abdominal view (VIIIt—abdominal tergite VIII, VIIs—abdominal sternite VII).

*Glyphina betulae* (Linnaeus, 1758) (Figure 3).

**Abdominal tergite VIII:** on the abdomen VIII from the cauda side, a row of massive setae is visible. They are placed every ten of micrometers. Segment surface with a delicate texture, dotted with cuticle protrusions.

**Cauda:** short but clearly distinguished, with the shape of a triangle with a strongly rounded tip. At the top, there is a pair of large setae. The cuticle is covered with single or multiple spinules, slender both at the base and at the apex.

**Anal plate:** wider than it is long, with a convex narrowing along the anus–vulva axis. Cuticle spinules present on the entire surface, the longest at the line of contact with the anal opening. Setae on AP densely distributed, the shortest, the most massive and the longest ones near the edges.

**Genital plate:** trapezoidal, approximately 300 μm wide, with shorter arms pointing towards the genital pore. Covered with delicate puncturing of the cuticle from its distal part to the more pronounced teeth of the cuticle at its apical part (near the APL). The two rows of setae are clearly marked: one at the apical part, the other running roughly through its center, evenly dense. Two additional setae nests are located at either end of the genital plate.

**Figure 4:**
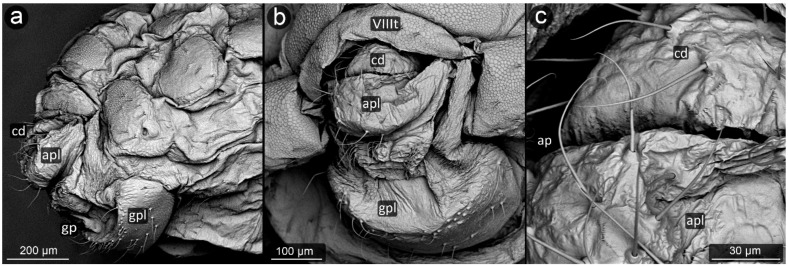
Perianal structure of *Prociphilus bumeliae* in SEM; (**a**)—lateral view, (**b**)—rear view, (**c**)—close-up of the cauda surface and anal plate.

*Prociphilus bumeliae* (Schrank, 1801) (Figure 4).

**Abdominal tergite VIII:** covered with wax glands.

**Cauda:** cuticle of cauda without wax glands, almost smooth with very few spiny protrusions on its surface. Cauda short and poorly defined, without a constriction in the base part, crescent-shaped, and narrow.

**Anal plate:** wide, more massive in its upper part, with setae bending towards anal pore. In the apical part, setae evenly spaced, all roughly the same length and diameter, including those on the cauda.

**Genital plate:** rectangular, with a smooth surface marked with setae in two of its areas; at its longer edge, at the point of contact with the anal pore, located more densely and in a more ordered line; the second setae line on the genital plate is wave-shaped in the mid-posterior part of the genital plate.

**Figure 5:**
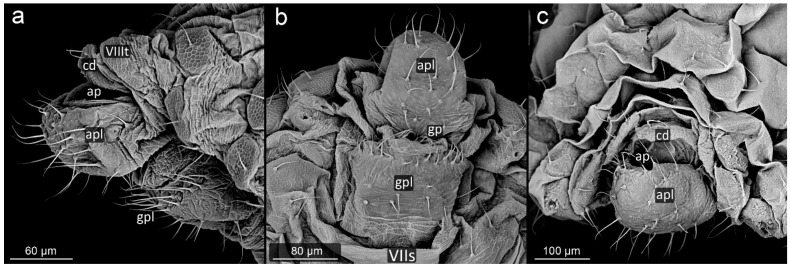
Perianal structure of *Prociphilus fraxini* in SEM; (**a**)—lateral view, (**b**)—abdominal view, (**c**)—rear view.

*Prociphilus fraxini* (Fabricius, 1777) (Figure 5)

**Abdominal tergite VIII:** wax glands visible on abdominal tergite VIII.

**Cauda:** cauda short, crescent-shaped, narrow.

**Anal plate:** The anal opening is situated upwards, the anal plate is protruding to the rear, convex. Its shape resembles a rectangle, massive. Its surface is sparsely covered with longer setae, bent towards anal pore. Cuticle of cauda very delicately serrated, spinules appearing as strips of short serrations on the surface of the cuticle.

**Genital plate:** large, square, very sparsely marked with cuticle outgrowths. Approximately 17–19 setae on its upper edge, additionally several in its middle part.

**Figure 6:**
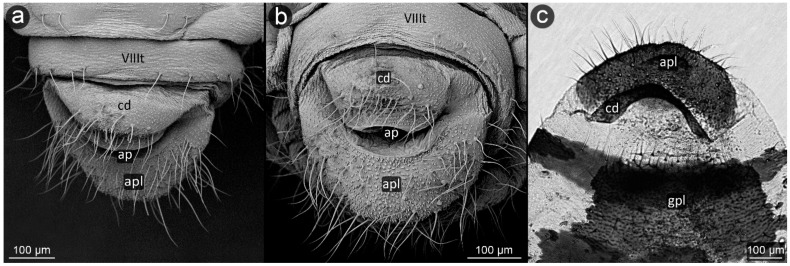
Perianal structure of *Symydobius oblongus*; (**a**)—dorsal view (SEM), (**b**)—rear view (SEM), (**c**)— rear view in light microscopy.

*Symydobius oblongus* (von Heyden, 1837) (Figure 6).

**Abdominal tergite VIII:** with a fine micro-relief typical for the rest of the body, with a row of setae at the outer edge of the tergite.

**Cauda:** short, poorly separated without narrowing in the basal part, very gently conical. The dorsal surface of the cauda without setae, delicately covered with micro-relief. Setae on cauda form a triangle with the apex at the tip of the caudal cone. A slight increase in the size of spinules in the microstructure of cuticle is noticeable over the entire surface of the apical part of cauda. There is an approximately 70 μm wide band at the base of the cauda devoid of setae, only with rows of spinules.

**Anal plate:** large, densely covered with setae of a wide range of sizes, covered with fine micro-relief of spinules and denticles.

**Genital plate:** large, square-shaped.

**Figure 7:**
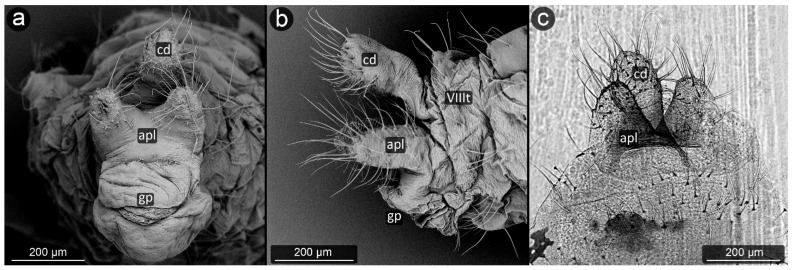
Perianal structure of *Panaphis juglandis*; (**a**)—rear view (SEM), (**b**)—lateral view (SEM), (**c**)— rear view in light microscopy.

*Panaphis juglandis* (Goeze, 1778) (Figure 7).

**Abdominal tergite VIII**: on the last abdominal segment, there is a row of densely arranged setae, of a length and thickness comparable to the remaining ones on the cauda and anal plate.

**Cauda:** elongated, on the dorsal surface devoid of setae, cuticle texture relatively smooth. On the ventral side of the cauda, there are many setae which become gradually shorter towards the anus and the cuticle is covered with small spinules.

**Anal plate:** anal plate strongly bilobed, with two separate lobes of the length comparable to cauda. Lobes covered with many long setae on whole perimeter. Spinules of cuticle only slightly marked at the top of each of the two lobes of the anal plate.

**Genital plate:** small, poorly sclerotized, elongated, much wider than it is long.

**Figure 8:**
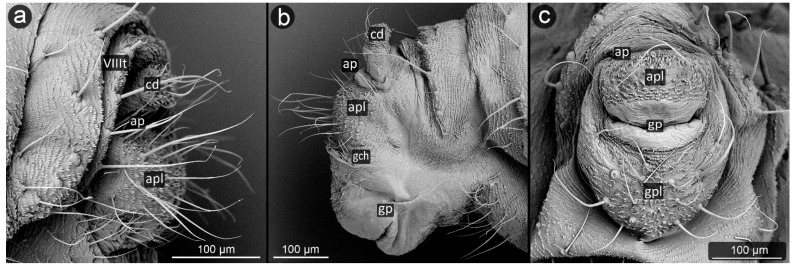
Perianal structure of *Chaitophorus nassonowi* in SEM; (**a**)—lateral view, (**b**)—lateral view, (**c**)—rear view (cauda [cd] differentiated inside the body).

*Chaitophorus nassonowi* Mordvilko, 1894 (Figure 8).

**Abdominal tergite VIII:** the micro-sculpture strengthens in the border belt at the cauda, where the row of long setae appears.

**Cauda:** small, knobbed, ca. 46 μm long, covered with cuticular spinules on whole apical part.

**Anal plate:** somewhat higher than it is wide, densely covered with protruding spinules having multiple finger-like processes. There are two types of setae on the anal plate—next to the anus, they are only delicate, straight and directed upwards, while below they are longer, set perpendicular to the plate.

**Genital plate:** very well developed and bears numerous long, fine and pointed setae.

**Figure 9:**
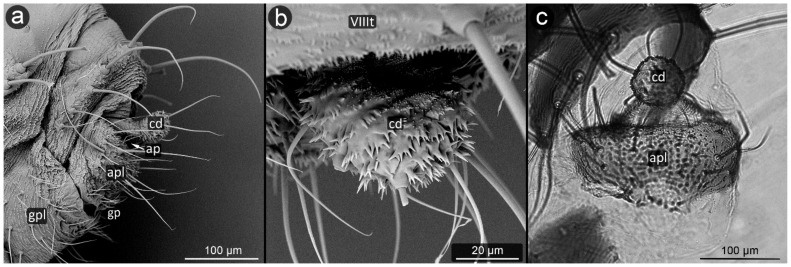
Perianal structure of *Chaitophorus populeti*; (**a**)—lateral view (SEM), (**b**)—close-up on the surface of cauda-protruding spinules (SEM), (**c**)—rear view in light microscopy.

*Chaitophorus populeti* (Panzer, 1801) (Figure 9).

**Abdominal tergite VIII:** with 6–7 very long, fine setae, bent towards cauda. The lateral setae of this tergite are significantly shorter.

**Cauda:** small and knobbed with a few fine setae at the top, cuticle with protruding spinules.

**Anal plate:** longer than it is wide, with long perpendicular setae. Surface covered with protruding spinules having multiple finger-like processes.

**Genital plate:** large, with a delicate micro-relief set in transverse stripes among which there are three rows of long, pointed setae, of which the distal one is positioned uppermost, bent towards the genital pore, while the setae of the remaining rows are perpendicular to the surface of the genital plate.

**Figure 10:**
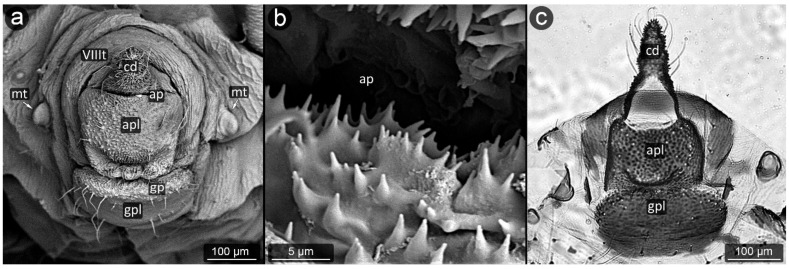
Perianal structure of *Aphis jacobaeae*; (**a**)—rear view (SEM), (**b**)—close-up on the surface of anal plate-protruding spinules (SEM), (**c**)— rear view in light microscopy.

*Aphis jacobaeae* Schrank, 1801 (Figure 10).

**Abdominal tergite VIII:** with a delicate texture of cuticle, having eight setae of medium length, four setae bent at the ends; longer ones at the top, decreasing their lengths toward the marginal sides.

**Cauda:** elongated, finger-like, tapering towards the apex, relatively long. Its entire surface is covered with a distinct microsculpture in the form of mainly single, maximum double-ended spinules. On both sides of the cauda, there are approximately 12–14 setae.

**Anal plate:** trapezoid, wider at the anal pore, with a distinct furrow separating the reproductive zone. The microsculpture is noticeably more delicate than that of the cauda, the spinules on its surface are low, rounded; finer, more densely located and a little sharper at distal edges. Setae on anal plate are short, completely absent in its central area.

**Genital plate:** large, strongly sclerotized, microsculpture visible only on the side adjacent to the pore genital, the remaining part is smooth. Setae in two lines at opposite parts of the plate: right next to the genital pore and at its proximal edge.

**Figure 11:**
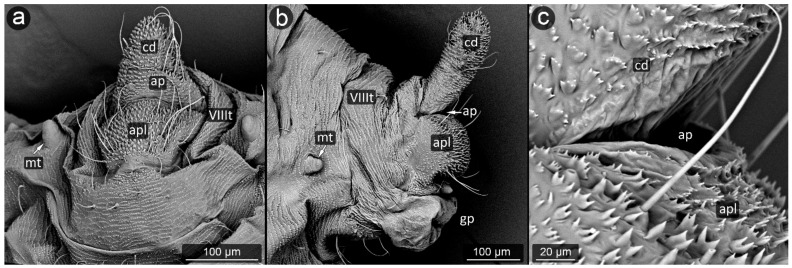
Perianal structure of *Aphis pomi* in SEM; (**a**)—rear–abdominal view, (**b**)—lateral view, (**c**)—close-up on the surface of anal plate and cauda-protruding spinules.

*Aphis pomi* De Geer, 1773 (Figure 11).

**Abdominal tergite VIII:** with dense, fine microsculpture, two short setae with pointed apices at the cauda base are present.

**Cauda:** long, finger-like, approximately 200 μm long. Spinules become more prominent closer to the apex and on the ventral side. Setae on the dorsal surface of cauda are thinner and shorter, while on the ventral part they are thicker and longer.

**Anal plate:** rectangular, cuticular spinules over the entire surface, smaller at the anal opening, elsewhere as prominent as at the apex of the cauda, single or forked. Setae on upper edge of anal plate bent towards the anus, run almost parallel to the cauda.

**Genital plate:** well developed with a few setae, of which the shorter and more delicate ones are located in its apical part near the genital pore, and the more massive ones with a massive base sheaths are located in its center at a short distance from each other.

**Figure 12:**
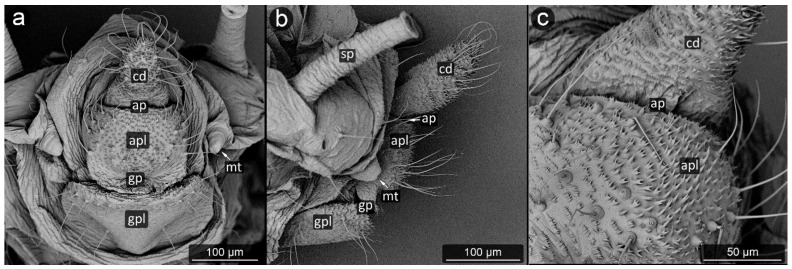
Perianal structure of *Aphis sedi* in SEM; (**a**)—rear view, (**b**)—lateral view, (**c**)—close-up on the surface of anal plate and cauda-protruding spinules; (sp—siphunculus).

*Aphis sedi* Kaltenbach, 1843 (Figure 12).

**Abdominal tergite VIII:** with a distinct cuticular microsculpture, with two setae in its apical part.

**Cauda**: setae located along the distal part of the cauda, towards the apex, latero-ventrally, with tips bent towards the mid axis of the body. Microsculpture with rows of spinules which become larger at apex and on ventral side.

**Anal plate:** square-shaped, densely covered with long and relatively massive spinules, longer along the central area towards the anus. Setae located mostly near the edges of the plate, in a semicircle, pointing upwards.

**Genital plate:** large, square-shaped, with a slight microsculpture over the entire surface, except for the strip of longer spinules of the cuticle at the genital pore. At the distal part near genital pore, two areas covered with long setae, placed at the margins; remaining part of plate with few long setae.

**Figure 13:**
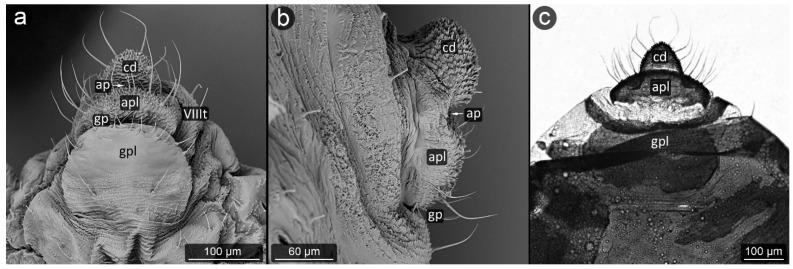
Perianal structure of *Brachycaudus tragopogonis*; (**a**)—abdominal view (SEM), (**b**)—lateral view (SEM), (**c**)—rear view in light microscopy.

*Brachycaudus tragopogonis* (Kaltenbach, 1843) (Figure 13).

**Abdominal tergite VIII:** with a row of six short, blunt setae, slightly capitate at the apices.

**Cauda:** short, rounded, poorly separated from the rest of the body, with only a slight constriction visible at its base. There are a few setae on the sides of the cauda, bent towards the mid-axis of the body. A well-defined microsculpture of cuticle with spinules in the shape of the dentate lobes covering the cauda surface, becoming more massive on the ventral side of the cauda.

**Anal plate:** rectangular, wider than it is long, with microsculpture of cuticle similar to that of cauda, covered with long setae bent towards the anus.

**Genital plate:** large and massive, rounded. The distal part of the genital plate with a smooth surface, with fine microsculpture at both poles. Setae are densely located in its apical part, pointed, pointing upwards (to the genital pore). Two long setae in the center of plate at its proximal part.

**Figure 14:**
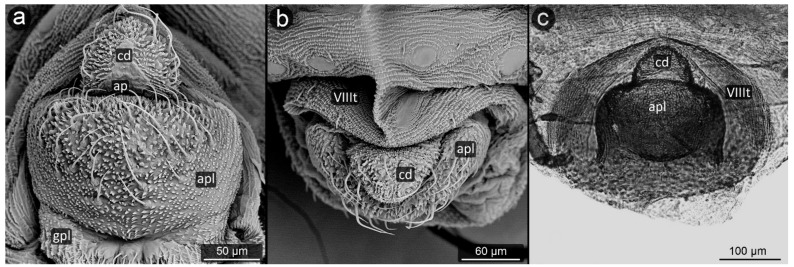
Perianal structure of *Anuraphis catonii*; (**a**)—rear view (SEM), (**b**)—dorsal view (SEM), (**c**)— rear view in light microscopy.

*Anuraphis catonii* Hille Ris Lambers, 1935 (Figure 14).

**Abdominal tergite VIII:** with a spinal tubercles in its dorsal part; several short, blunt setae at the distal edge.

**Cauda:** small, triangular, with dense and prominent microsculpture and with two strands of setae placed laterally.

**Anal plate:** setae unevenly arranged in the central part of the plate. The cuticle of both cauda and anal plate significantly sculptured, with spinules the more massive and protruding the closer to the center of the anal plate and latero-ventral part of cauda.

**Genital plate:** well developed, covered with dense microsculpture and bears numerous short, fine setae.

**Figure 15:**
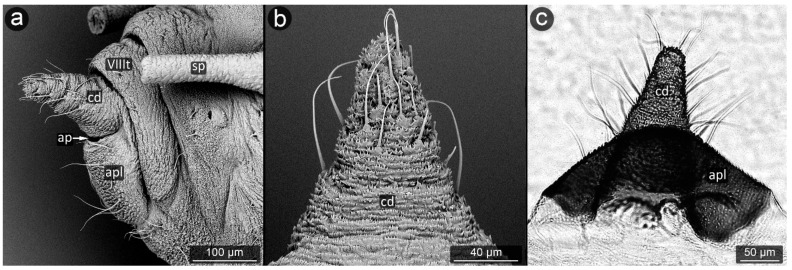
Perianal structure of *Metopeurum fuscoviride*; (**a**)—lateral view (SEM), (**b**)—close-up of cauda (SEM), (**c**)—rear view in light microscopy.

*Metopeurum fuscoviride* Stroyan, 1950 (Figure 15).

**Abdominal tergite VIII:** cuticular microsculpture dense, with transverse rows of spinules; setae very short, blunt, not reaching cauda.

**Cauda:** long, conical in shape. The cuticle surface is densely microsculptured, covered with rows of spinules, more prominent on the ventral surface. Setae on cauda are long, bent towards its apex.

**Anal plate:** densely covered with spinules, especially in the midline and covered with long setae.

**Genital plate:** well developed, trapezoidal in shape with a distal margin at the genital pore shorter; microsculpture dense but delicate, with short setae, especially at the genital pore.

**Figure 16:**
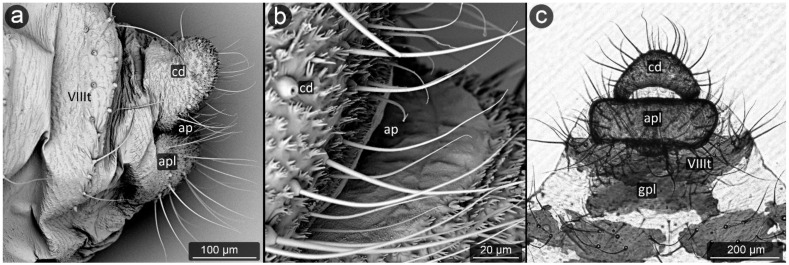
Perianal structure of *Pterocomma konoi*; (**a**)—lateral view (SEM), (**b**)—close-up of anal pore and cauda (SEM), (**c**)— rear view in light microscopy.

*Pterocomma konoi* Hori, 1939 (Figure 16).

**Abdominal tergite VIII:** with a very fine microsculpture, with setae arranged in two rows.

**Cauda:** short, rounded, covered with dense microsculpture of prominent spinules, especially in its ventral surface. Setae on cauda long, bent toward the mid axis of body.

**Anal plate:** rectangular, wider than it is long. Setae on anal plate short and thinner at the entrance to the anus, long and gently raised upwards in the further parts of the plate. Microsculpture most prominent below the anal pore in form of rows of spinules.

**Genital plate:** very well developed, crescent-shaped and bears numerous long, fine, and pointed setae.

**Figure 17:**
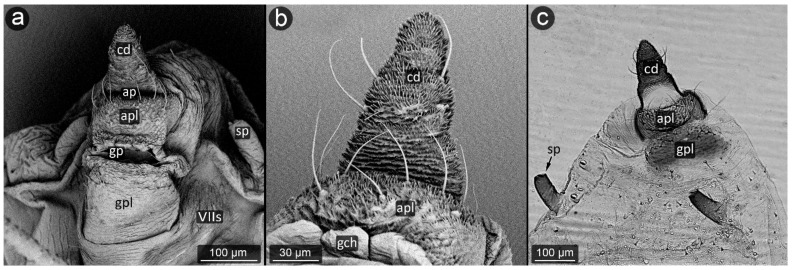
Perianal structure of *Semiaphis dauci*; (**a**)—rear view (SEM), (**b**)—close-up of cauda (SEM), (**c**)— rear view in light microscopy.

*Semiaphis dauci* (Fabricius, 1775) (Figure 17).

**Abdominal tergite VIII:** with a very fine microsculpture, with several (2–3) very short, blunt setae along the distal margin.

**Cauda:** medium length, slightly tapering to the tip, conical, covered with few long and curved setae. Cuticular spinules positioned vertically to the surface of the cauda on the ventral side, dorsal sculpture less prominent.

**Anal plate:** clearly rectangular in shape, wider than it is long, densely covered with cuticular spinules all over the central surface and with few fine setae placed latero-distally.

**Genital plate:** rectangular, large and well sclerotized. In its distal part (near the genital pore), there are thick setae with rounded ends.

**Figure 18:**
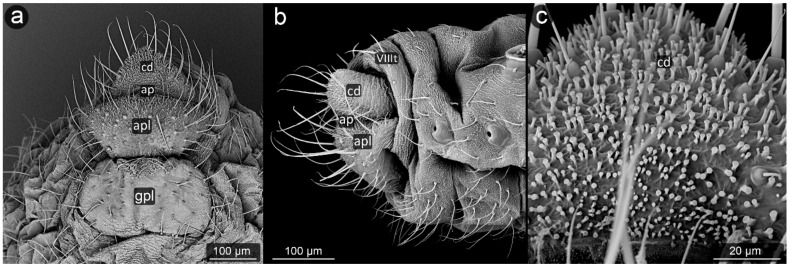
Perianal structure of *Cinara pini* in SEM; (**a**)—rear view, (**b**)—lateral view, (**c**)—close-up on the surface of cauda-protruding spinules.

*Cinara pini* (Linnaeus, 1758) (Figure 18).

**Abdominal tergite VIII:** fine cuticular microsculpture observed over the entire surface of the segment. Setae lined up in a row at the edge of the cauda. Setae located symmetrically at equal distances, always directed towards the long axis of the body (to the tip of the cauda), pointed.

**Cauda:** short, broadly rounded, its dorsal surface very marked, with major imbrication. The ventral side covered with long, fine setae, bent towards the anus. Cuticular microsculpture very specific, in the form of unique pin-shaped cuticle appendages with a flattened head, densely covering the ventral part of the cauda and also the upper edge of the anal plate at the anus.

**Anal plate:** a trapezoidal with a longer edge near the anus, covered with setae of various lengths, longer near the anal pore, bent towards the anus.

**Genital plate:** rectangular, with a slight narrowing in the center, clearly rounded margins, wider than it is long. Central part with delicate microsculpture; setae near the genital pore bent towards it; straight at the margins.

**Figure 19:**
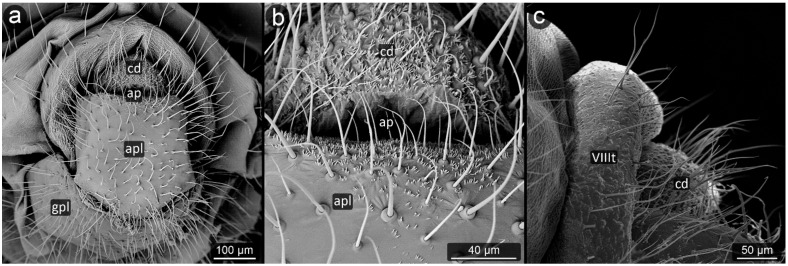
Perianal structure of *Lachnus pallipes* in SEM; (**a**)—rear view, (**b**)—close-up on the surface of cauda and anal plate, (**c**)—lateral view.

*Lachnus pallipes* (Hartig, 1841) (Figure 19).

**Abdominal tergite VIII:** the last segment of the abdomen with a smooth surface, covered with approximately 30 pointed setae. Distal margin with dense but delicate microsculpture.

**Cauda:** very small, rounded, densely setose. There are two lengths of setae on this structure: in the middle, shorter setae form a triangle; on the sides, a two-row strip of longer setae. Both types of setae are pointed. Cauda cuticle with distinct microsculpture in form of protruding, pointed spinules with multiple tips.

**Anal plate:** large, the pattern of cuticular spinules covers only the area near anal pore, forming a delicate triangle pattern with the apex heading towards the genital plate, the rest of the surface is smooth. The anal plate has the shape of an inverted isosceles trapezoid with an angle of 90 degrees at the base, densely covered with setae on the entire surface.

**Genital plate:** well developed, smooth, covered by very short and pointed setae.

**Figure 20:**
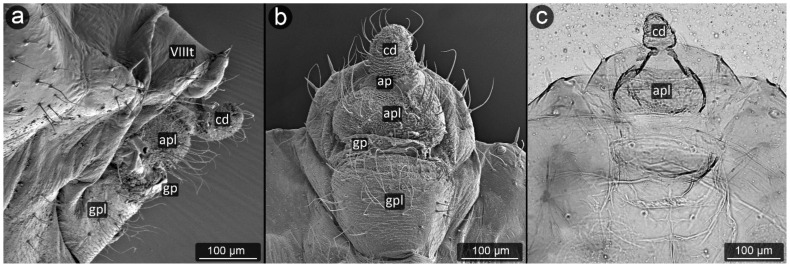
Perianal structure of *Thelaxes dryophila*; (**a**)—lateral view (SEM), (**b**)—rear view (SEM), (**c**)—rear view in light microscopy.

*Thelaxes dryophila* (Schrank, 1801) (Figure 20).

**Abdominal tergite VIII:** with a smooth surface, with a row of long, pointed setae on the distal margin.

**Cauda:** short but well separated, knobbed, slightly bent upwards. Cuticular microsculpture on the entire surface of the cauda take the form of rows of small spinules, relatively slightly structured, parallel to the anal opening.

**Anal plate:** rectangular, wider than it is long. Setae on both sides of the plate facing cauda. In the center of the plate, there is a shallow groove on which no setae protrude; the whole plate is covered with cuticular spinules structured similar as on the cauda.

**Genital plate:** well developed, square in outline, with dense cuticle processes in the genital part, and very delicate carving in the rest. Long setae all over its surface.

### 3.2. Facultatively Myrmecophilous Aphids

**Figure 21:**
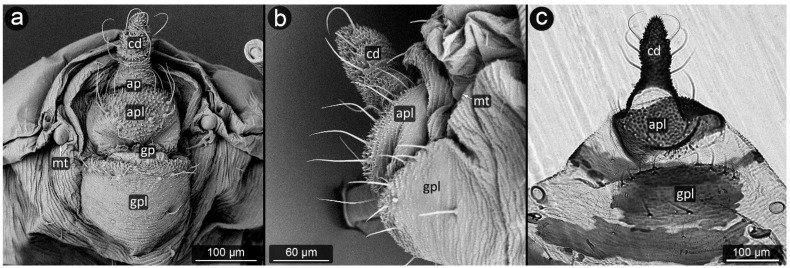
Perianal structure of *Aphis craccivora*; (**a**)—rear view (SEM), (**b**)—lateral view (SEM), (**c**)— rear view in light microscopy.

*Aphis craccivora* Koch, 1854 (Figure 21).

**Abdominal tergite VIII:** covered with transverse rows of minute spinules, short setae with a slightly capitate apices along the distal margin.

**Cauda:** with visible cuticular spinules, becoming more massive closer to the apex. Cauda long, with three-four setae on each side, bent towards the anus.

**Anal plate:** covered with cuticular spinules, in shape of an inverted triangle. Setae located on the lateral edges of the anal plate, not directly below the anal pore.

**Genital plate:** at the genital pore also covered by several rows of spinules and a single row of setae.

**Figure 22:**
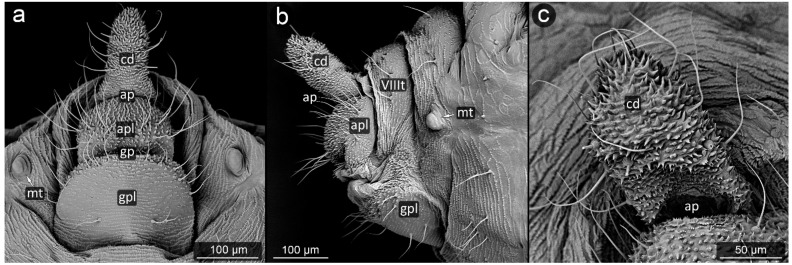
Perianal structure of *Aphis fabae* in SEM; (**a**)—rear view, (**b**)—lateral view, (**c**)—close-up on the surface of cauda and anal plate.

*Aphis fabae* Scopoli, 1763 (Figure 22).

**Abdominal tergite VIII:** covered with transverse rows of minute spinules, in the center a row of long and pointed setae directed towards the cauda.

**Cauda:** medium-sized, finger-shaped. Covered with dense cuticular microsculpture of spinules taking the form of sharpened denticles, the longest at the apex. Setae located on lateral and ventral parts of cauda, slightly bent towards long axis of body.

**Anal plate:** square-shaped, rounded at the genital part. Almost entire surface of anal plate covered with prominent cuticular spinules, mostly single, but at the anal pore forming clusters with a few denticles in a single cluster. Setae mostly on the sides of the plate, with pointed apices, slightly bent towards anal pore.

**Genital plate:** well developed, slightly oval in outline, setae occurring mainly in its lateral and distal parts; at the line of contact with the genital pore, five long, pointed setae, set upwards, in its distal part on both sides.

**Figure 23:**
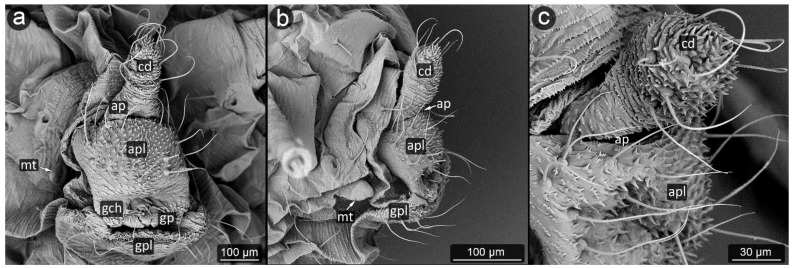
Perianal structure of *Aphis hederae* in SEM; (**a**)—rear view, (**b**)—lateral view, (**c**)—close-up on the surface of cauda and anal plate; (gch—gonochaetae).

*Aphis hederae* Kaltenbach, 1843 (Figure 23).

**Abdominal tergite VIII:** covered with transverse rows of minute spinules; two setae on both sides.

**Cauda:** clearly elongated, with a set of long setae on the sides, bent towards the anus. Cuticular spinules present all over the cauda, but are clearly more massive and longer on the ventral side and at the apex.

**Anal plate:** square-shaped, with mostly single, prominent spinules all over its surface; long setae on whole surface of the plate.

**Genital plate:** large and well developed, cuticular microsculpture in the form of spinules becomes denser at the opening of the genital pore; at the same edge, there is a single row of long, pointed setae.

**Figure 24:**
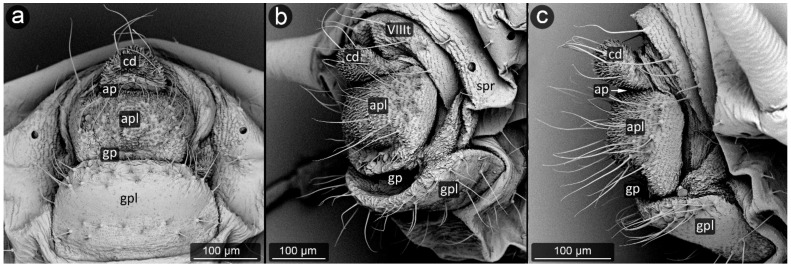
Perianal structure of *Brachycaudus cardui* in SEM; (**a**)—rear view, (**b**,**c**)—lateral view; (spr—spiracle).

*Brachycaudus cardui* (Linnaeus, 1758) (Figure 24).

**Abdominal tergite VIII:** cuticular microsculpture in the form of imbricated spinules; six long setae at equal intervals from each other, bent towards cauda, five to six times longer than the setae on the remaining segments of the abdomen.

**Cauda:** short and rounded with dense cuticular microsculpture in the form of spinules; with a few long and bent setae, mainly at the apex.

**Anal plate:** relatively large, rectangular and covered with cuticular spinules and with many long setae.

**Genital plate:** large and rectangular, with smooth cuticle except for distal and proximal margins, where small spinules and long setae are present.

**Figure 25:**
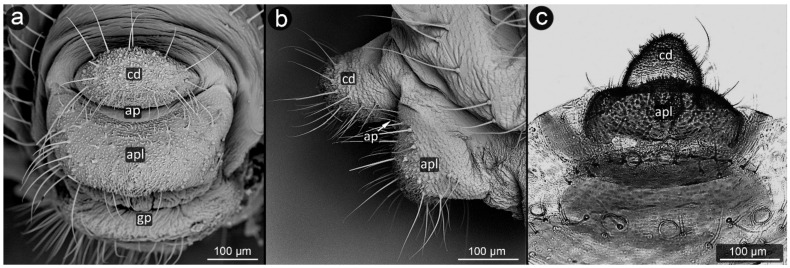
Perianal structure of *Dysaphis anthrisci*; (**a**)—rear view (SEM), (**b**)—lateral view (SEM), (**c**)— rear view in light microscopy.

*Dysaphis anthrisci* Börner, 1950 (Figure 25).

**Abdominal tergite VIII:** smooth surface and a strip of long, pointed setae in the middle of the tergite.

**Cauda:** short and broadly rounded. Distal half on cauda covered with cuticular spinules. Long, pointed setae embedded quite densely on its entire surface, of which delicate and short are located only on the ventral side, in the vicinity of the anus.

**Anal plate:** wide, the upper corners join with abdominal tergite VIII to form a semi-circular sinus beneath cauda in which the anus is located. There are 30–35 small, very short setae in the central zone, distinct from strands of longer setae on the sides. Both cauda and anal plate are covered with relatively weak microsculpture in form of short spinules, more prominent on cauda.

**Genital plate:** large, rectangular in shape, well sclerotized, with a large number of setae at both margin, of which the setae at genital pore are perpendicular to the plate.

**Figure 26:**
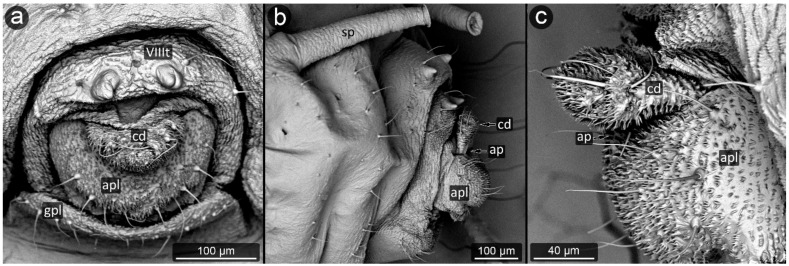
Perianal structure of *Dysaphis plantaginea* in SEM; (**a**)—rear view, (**b**)—lateral view, (**c**)—close-up on the surface of cauda and anal plate.

*Dysaphis plantaginea* (Passerini, 1860) (Figure 26).

**Abdominal tergite VIII:** with a delicate microsculpture and two spinal tubercles in the middle of the segment, coming directly from the cuticle. Few long setae bent towards the cauda.

**Cauda:** short, rounded, covered with a dense and prominent microsculpture and a few fine, bent setae.

**Anal plate:** small, rectangular; the long, fine setae form a semi-circle along the lower edge of the plate, which is also covered densely with prominent, sharp cuticular spinules.

**Genital plate:** well developed, rectangular with rounded edges, with fine setae and cuticular spinules near the genital pore.

**Figure 27:**
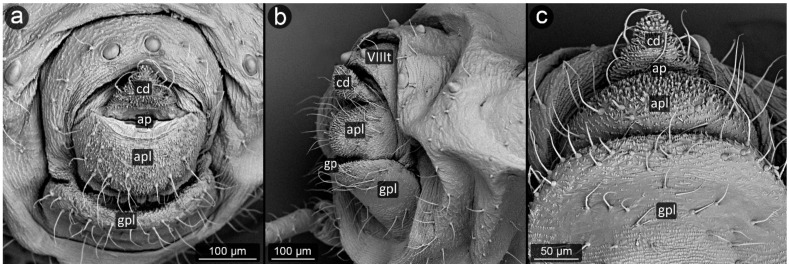
Perianal structure of *Dysaphis sorbi* in SEM; (**a**)—rear view, (**b**)—lateral view, (**c**)—abdominal view.

*Dysaphis sorbi* (Kaltenbach, 1843) (Figure 27).

**Abdominal tergite VIII:** two smooth-surfaced spinal tubercles present in the central part of the segment. On the same transverse axis, there is a row of long and pointed setae.

**Cauda:** quite short, but well separated, similar in shape to an equilateral triangle. The cuticular microsculpture is well developed, with many prominent spinules on its entire surface, but especially at the apex. On the cauda few long, bent setae on both sides, absent from the middle part close to the anus.

**Anal plate:** microsculpture of the cuticle on the anal plate is similar to cauda, all spinules are of the same height, with three to five processes. Setae on anal plate densely distributed and long, arranged in a three-row semicircle around the anus. Area closer to anal pore devoid of setae.

**Genital plate:** large rectangular, well sclerotized, with many but short setae and weak microsculpture, stronger only at the distal margin.

**Figure 28:**
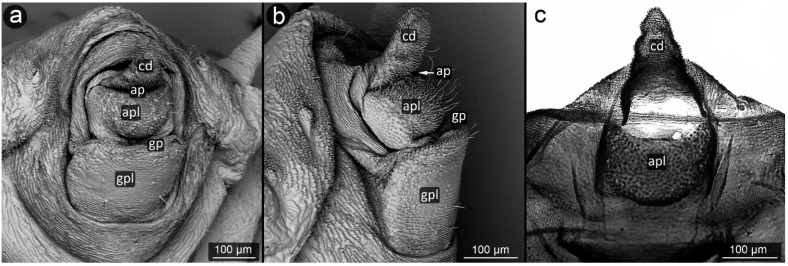
Perianal structure of *Myzus cerasi*; (**a**)—rear view (SEM), (**b**)—lateral view (SEM), (**c**)— rear view in light microscopy.

*Myzus cerasi* (Fabricius, 1775) (Figure 28).

**Abdominal tergite VIII:** cuticular microsculpture in the form of transverse rows of small spinules; there are few, very short and thin setae.

**Cauda:** elongated, triangular with rounded apex; covered with dense cuticular microsculpture, more prominent on its ventral side, and a few fine setae on each side.

**Anal plate:** rectangular, almost square-shaped, also densely covered with prominent cuticular spinules and fine setae.

**Genital plate:** square-shaped in outline, with weak microsculpture, stronger only at the margins; in the distal margin, there is a row of short, rounded setae; on the rest of the plate, single setae of the same structure.

**Figure 29:**
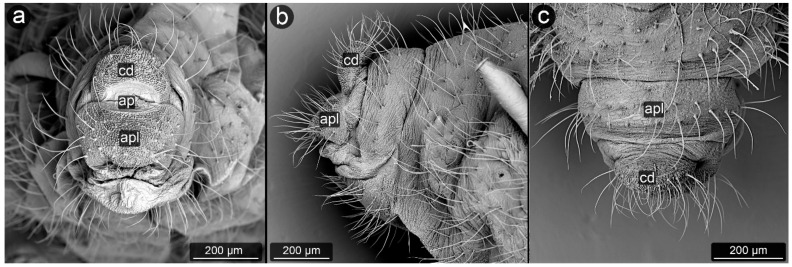
Perianal structure of *Pterocomma rufipes* in SEM; (**a**)—rear view, (**b**)—lateral view, (**c**)—dorsal view.

*Pterocomma rufipes* (Hartig, 1841) (Figure 29).

**Abdominal tergite VIII:** setae arranged in two rows, a total of a dozen.

**Cauda:** large, flat, triangular in outline with strongly rounded angles, with the apex on the side of the ridge. Setae occur all over its surface, approximately 22–25 in total, shorter and more delicate than on segment eight (approximately 40 μm). Microtrichium clearly visible on the whole surface, the longest in its central part, cauda broadly tongue-shaped.

**Anal plate:** large, semicircular, densely covered with setae of various lengths. Microtrichium present but softer than cauda.

**Genital plate:** well developed, rectangular and massive.

**Figure 30:**
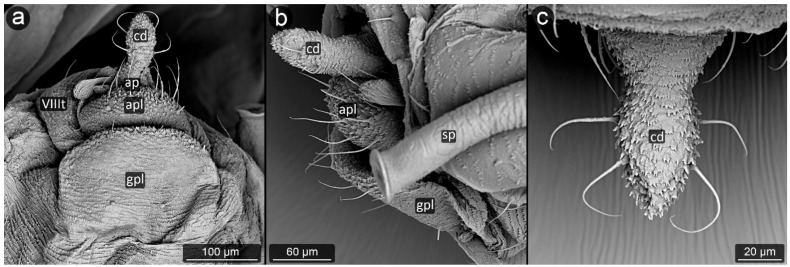
Perianal structure of *Rhopalosiphum padi* in SEM; (**a**)—abdominal view, (**b**)—lateral view, (**c**)—close-up of cauda.

*Rhopalosiphum padi* (Linnaeus, 1758) (Figure 30)

**Abdominal tergite VIII:** with a delicate micro-relief of the epidermis. Several setae distributed throughout his ministry.

**Cauda:** tassel-shaped, with four long setae on its apical part. The microsculpture is present on the entire surface of the cauda, serrated.

**Anal plate:** entire, wider than longer, strongly convex in the middle parts, where the microsculpture is clearly more massive. Setae only in the central area of it, forming a basket with apical parts of the set facing the anus.

**Genital plate:** large, with an oval side at the pore genital, where there is also a narrow strip of microsculpture running along its entire edge, with a setae strip on its edge. Setae is slightly shorter than the anal plate, which is an extension of its basket.

## 4. Discussion

Presented results showed that there were only minute differences in structure of perianal region between facultatively and obligatory myrmecophilous aphids. The existing differences may be traced in two aspects of aphid morphology: relationship between abdominal segments and surface of cuticle.

### 4.1. Cauda and Anal Plate

Although the commonly accepted paradigm is that obligatorily myrmecophilous aphids have shortened cauda [27], results of our study indicate that obligatory myrmecophiles have the cauda longer in relation to its width (Table 1). This means they have slender, more elongated cauda than facultative myrmecophiles, cauda of which seems to be as long as it is wide, e.g., *Aphis jacobaeae* vs. *A. fabae*. The reason for such result may be either inappropriate assignment of studied species to category of myrmecophily, or that this morphological trait at these levels of difference in myrmecophily is not significant for trophobionts. Theoretically, shorter cauda is facilitation for ants to remove droplet of honeydew, but the example of *Macrosiphoniella yomogicola* (Matsumura, 1917) shows that long cauda is not an obstacle to obligatory myrmecophily [53]. Likewise, *Aphis pomi* (Figure 11) or *A. sedi* (Figure 12) have long cauda and are ant-attended.

In addition, anal plate seems to be of no particular shape in case of obligatory and facultative myrmecophiles, although in case of Aphidinae it was significantly higher than it was wide in facultative species, e.g., *A. jacobaeae* vs. *A. fabae* (Table 1). This result seems odd as it is believed that higher anal plate is indicative of adaptation to myrmecophily (Depa et al. 2020), serving to keep honeydew droplet until removed by ant. This case concerned subterranean species studied by Zwölfer [32,33,34,35] and repeated by Kanturski et al. [31], where this trait seemed to follow obligatory myrmecophily. However, all these species are subterranean, living on underground parts of plants or in ant chambers, covered by soil (e.g., *Stomaphis* spp.) [36]. In addition, some of them spend part of life cycle in galls (e.g., Fordini) [54], where they are not ant-attended but may require special perianal adaptations to remove and avoid droplet of honeydew [27]. Such life modes create very specific conditions, where either gall microclimate or soil microbiota and honeydew pose increased danger for aphid survival and such shape of anal plate might have evolved to facilitate honeydew removal and was an exaptation for mutualistic relationship with ants. While this thesis requires further studies, presented results of obligatory myrmecophilous aphids put in question the modifications of cauda and anal plate as adaptations to trophobiosis. It seems that these features are rather related with general morphology of whole tribes and subfamilies than with adaptation to mutualism with ants of particular species. It may be hypothesized that environmental conditions following the adaptation to host plant are more influential on morphology of perianal regions than mutualism with ants. The latter might have developed multiple times within single genus, e.g., *Chaitophorus* [55], while the relation with host plant and its habitat seems to be more stable. While myrmecophilous coccids also present some homologous morphological modifications in reference to ant attendance in domatia, e.g., more setae around anal pore, as well as reduced anal tubes [56], other sternorhynchans, e.g., psyllids even if ant-attended, still cover their honeydew droplet with wax due to the presence of circumanal ring of setae [57]. Therefore, it seems that perianal modifications to protect the insect from contamination by honeydew in various feeding locations is evolutionary, older adaptation than to mutualism with ants.

### 4.2. Cuticle Surface and Setae of the Perianal Area

The body of the aphid has a fairly delicate cuticle, sometimes strengthened with smaller or larger patches of sclerites, but they rarely cover the entire body. Their main function is protection against environmental conditions and the thickness of its individual layers varies depending on the place of occurrence [58]. Cuticle of aphids is very often covered with microrelief creating variable processes, spinules and wrinkles.

During the study, significant differences in morphology of cuticle were observed in various aphid species, from a completely smooth surface (e.g., in *Panaphis juglandis*), through selectively carved (*Lachnus pallipes*) to densely carved microsculpture covering the entire perianal region (*Dysaphis anthrisci, Cinara pini*) (Figure 18 and Figure 25). What is more, the forms created by cuticular processes also took on various sizes and forms depending on the species and location on perianal area. Most often, the most shapely cusps were on the cauda and at apical part of the anal plate, next to the anus, or evenly covered the entire region with spinules or denticles of similar size and shape. The unusual shape of the cuticular spinules was observed in *Cinara pini* (Figure 18), where the underside of the cauda and the anal plate were covered with microrelief in the shape of single, mushroom-like appendages with flat apices. Such processes occurred in both larvae and adults, alate and apterous. It may be assumed that the specific shape is to increase the contact surface with the honeydew droplet, which will result in better stabilization.

The aphids studied have fine, sharp setae, many of these seem to be mechanoreceptors (trichoid sensilla) in perianal structures [31]. However, several types could be distinguished, mainly on the basis of their length and diameter. It seems that the type of setae in the anal area is not accidental, because even if on other parts of the body there are setae of various structure, e.g., spatulate setae [58] in *Chaitophorus populeti*, within perianal structures they always occur in one form in all investigated species. Although setae “typical” of species may occur on the anal plate, they have been noticed only in the anal region of the anal plate (e.g., *Geoica utricularia*) [31]. Furthermore, in all studied species, perianal setae are long and bent towards the anus. In case of caudal setae, they are usually positioned along the lateral midline and on ventral part of cauda (Figure 18a, Figure 23a and Figure 30c). Therefore, it may be also assumed that in all cases they serve to keep droplet of honeydew in position far from body. No significant difference in length or shape of the perianal setae between facultative and obligatory myrmecophilous species might be observed, but their shape and arrangement are similar to that of so far studied underground living species [31,36].

Presented results strongly suggest that the structure of cuticle may play the leading role in adaptation of aphids to mutualism with ants, as a general adaptation to managing potentially lethal droplet of honeydew. This subject has not been studied in aphids so far but it seems that microrelief of aphid cuticle may have additional hydrophobic effect on the honeydew droplet, kept in position by set of perianal setae, by creating the so-called “lotus effect”. This is a known phenomenon in nature, where the microspinules on the surface of living organism, either plant or animal, increase hydrophobic properties of this surface [59,60]. The presence of cuticular waxes was so far considered as a key factor preserving aphids in drowning in own honeydew [27], but this seems to be not the only agent in this mechanism. Not all aphids have significant waxy covering, and it is believed that myrmecophilous aphids have reduced wax cover. In case of aphids poorly covered with wax, it may be the microstructure of cuticle that keeps any moisture far from body, and this may also concern subterranean or underground living species. Existence of highly spiculose perianal tubercles in obligatorily myrmecophilous aphid genus *Stomaphis*, additionally conducting cryptic life mode, may contribute to drawing such thesis [36]. The extent of importance of cuticular microrelief in keeping honeydew droplet far from body but still close enough not to lose it until ant attendance requires further confirmation by comparison with non-myrmecophilous aphid species (Kaszyca-Taszakowska *in prep*.).

## Figures and Tables

**Table 1:** Aphid species studied with accordant results of ratios in their perianal structures (the mean values of series which differ statistically, *p* < 0.05, are in bold; below the table are explanations of the meaning of obtained values) (apl/cl—anal plate length to cauda length ratio, apl/apw—anal plate length to anal plate width ratio, cl/cw—cauda length do cauda width ratio, cw/chw—cauda width to cauda half width ratio; x—value of ratio).

Subfamily	Species		om	fm	om	fm	om	fm	om	fm
	obligatorily myrmecophilous (om)	facultatively myrmecophilous (fm)	apl/cl		apl/apw		cl/cw		cw/chw	
Thelaxinae	*Glyphina betulae* (Linnaeus, 1758)		0.43		0.26		1.27		1.89	
	*Thelaxes dryophila* (Schrank, 1801)		0.63		0.41		1.08		0.56	
Eriosomatinae		*Prociphilus bumeliae* (Schrank, 1801)		4.23		0.67		0.54		0.80
		*Prociphilus fraxini* (Fabricius, 1777)		2.37		1.19		0.78		0.73
Calaphidinae	*Symydobius oblongus* (von Heyden, 1837)		2.36		1.74		0.53		0.81	
	*Panaphis juglandis* (Goeze, 1778)		1.09		1.39		2.31		0.52	
Chaitophorinae	*Chaitophorus nassonowi* Mordvilko, 1894		2.51		2.37		1.38		0.52	
	*Chaitophorus populeti* (Panzer, 1801)		1.73		2.43		1.91		0.41	
Aphidinae	*Aphis acetosae* Linnaeus, 1761	*Aphis craccivora* Koch, 1854	0.67	1.28	0.61	2.01	2.44	1.93	0.58	0.75
	*Aphis jacobaeae* Schrank, 1801	*Aphis fabae* Scopoli, 1763	0.77	1.16	0.97	1.86	2.41	1.42	0.56	0.63
	*Aphis pomi* De Geer, 1773	*Aphis hederae* Kaltenbach, 1843	0.98	1.18	1.79	1.82	2.69	1.68	0.63	0.58
	*Aphis sedi* Kaltenbach, 1843	*Rhopalosiphum padi* (Linnaeus, 1758)	0.59	0.98	0.57	0.64	2.38	1.14	0.77	0.54
	*Anuraphis catonii* Hille Ris Lambers, 1935	*Brachycaudus cardui* (Linnaeus, 1758)	2.63	1.74	1.72	0.92	0.73	0.74	0.69	0.73
	*Brachycaudus tragopogonis* (Kaltenbach, 1843)		1.08		0.42		0.81		0.80	
	*Metopeurum fuscoviride* Stroyan, 1950	*Dysaphis anthrisci* Börner, 1950	0.96	2.18	0.92	2.31	1.46	0.94	0.51	0.80
		*Dysaphis plantaginea* (Passerini, 1860)		2.01		2.52		0.97		0.70
	*Semiaphis dauci* (Fabricius, 1775)	*Dysaphis sorbi* (Kaltenbach, 1843)	0.62	1.55	0.52	1.48	1.96	0.87	0.62	0.60
		*Myzus cerasi* (Fabricius, 1775)		1.15		1.81		1.39		0.56
	*Pterocomma konoi* Hori, 1939	*Pterocomma rufipes* (Hartig, 1841)	2.42	2.53	2.11	2.73	0.83	0.57	0.77	0.70
Lachninae	*Cinara pini* (Linnaeus, 1758)		1.76		1.63		0.61		0.68	
	*Lachnus pallipes* (Hartig, 1841)		3.00		2.13		0.61		0.82	
		mean for all samples:	1.43	1.86	1.29	1.66	**1.49**	**1.08**	0.71	0.68
		mean for Aphidinae	1.19	1.58	**1.07**	**1.81**	**1.75**	**1.17**	0.66	0.66
			x > 1—anal plate longer than cauda			x > 1—cauda longer than its base				
					x > 1—anal plate longer than wide				x < 1—cauda is narrowing towards apex	

## Data Availability

Data are contained within the article or Supplementary Materials.

## Supplementary Materials

The following supporting information can be downloaded at: https://www.mdpi.com/article/10.3390/insects13121160/s1, Table S1: Measurements of studied specimens; Table S2: Mean values of measured structures; Table S3: Mean ratios of measured structures.
