# Supplementary material for: Comparative Studies of Perianal Structures in Myrmecophilous Aphids (Hemiptera, Aphididae)
Source: Insects. 2022 Dec 16;13(12):1160. doi: 10.3390/insects13121160 (PMC9781728; doi:10.3390/insects13121160)
Supplement: Supplementary file 1 [file insects-13-01160-s001.zip › Supplementary Table S1.pdf]

Table S1: Measurements of studied specimens [mm].

Species	Anal plate length (mm)	Anal plate width in the widest place	Cauda length	Width cauda at the base	Half width cauda
Glyphina betulae	0.229	0.071	0.139	0.062	0.121
	0.309	0.068	0.185	0.074	0.149
	0.211	0.071	0.136	0.064	0.114
	0.225	0.059	0.135	0.051	0.095
	0.230	0.050	0.140	0.055	0.100
Prociphilus bumeliae	0.255	0.391	0.106	0.253	0.169
	0.258	0.388	0.080	0.194	0.162
	0.272	0.398	0.092	0.200	0.187
Prociphilus fraxini	0.422	0.363	0.124	0.317	0.189
	0.279	0.231	0.113	0.139	0.129
	0.292	0.237	0.113	0.158	0.129
Symydobius oblongus	0.403	0.147	0.107	0.297	0.240
	0.384	0.120	0.115	0.326	0.296
	0.371	0.092	0.105	0.317	0.232
	0.142	-	0.148	0.314	-
	0.158	0.310	0.142	0.202	0.170
Panaphis juglandis	0.372	0.258	0.325	0.260	0.136
	0.298	0.243	0.300	0.260	0.127
	0.303	0.201	0.268	0.220	0.123
Chaitophorus nassonowi	0.251	0.085	0.110	0.145	0.080
	0.222	0.097	0.091	0.125	0.052
	0.320	0.135	0.102	0.143	0.070
	0.239	0.100	0.104	0.138	0.084
	0.265	0.130	0.109	0.166	0.089
Chaitophorus populeti	0.212	0.101	0.098	0.142	0.065
	0.203	-	0.144	0.176	0.073
	0.184	0.094	-	-	0.054
	0.193	0.069	0.094	0.125	0.058
	0.279	-	0.158	0.187	0.074
Aphis acetosae	0.121	0.227	0.237	0.154	0.070
	0.150	0.215	0.225	0.139	0.079
	0.133	0.243	0.215	0.168	0.105
	0.133	0.199	0.150	0.123	0.069

	0.118	0.194	0.146	0.100	0.760
Aphis jacobaeae	0.190	0.141	0.110	0.195	0.090
	0.196	0.142	0.171	0.107	0.071
	0.184	0.116	0.207	0.120	0.062
	0.174	0.149	0.181	0.102	0.057
	0.253	0.130	0.183	0.103	0.074
Aphis pomi	0.209	0.128	0.240	0.118	0.089
	0.170	0.103	0.148	0.098	0.055
	0.140	0.085	0.154	0.090	0.049
	0.186	0.083	0.168	0.129	0.078
	0.188	0.102	0.202	0.100	0.068
Aphis sedi	0.146	0.206	0.233	0.113	0.084
	0.082	0.176	0.155	0.084	0.084
	0.092	0.150	0.145	0.080	0.054
	0.075	0.145	0.129	0.074	0.055
	0.061	0.119	0.115	0.074	0.049
Brachycaudus tragopogonis	0.206	0.098	0.089	0.118	0.096
	0.193	0.088	0.086	0.121	0.100
	0.219	0.078	0.075	0.115	0.097
	0.197	0.093	0.065	0.112	0.091
	0.209	0.070	0.078	0.139	0.100
Anuraphis catonii	0.254	-	0.107	0.128	0.089
	0.207	0.134	-	0.110	0.075
Metopeurum fuscoviride	0.256	0.138	0.237	0.155	0.074
	0.274	0.155	0.236	0.161	0.096
	0.268	0.317	0.257	0.183	0.085
	0.137	0.250	-	-	-
Pterocomma konoi	0.400	0.225	0.141	0.246	0.200
	0.307	0.165	0.136	0.251	0.187
	0.380	0.140	0.174	0.225	0.186
	0.342	0.158	0.129	0.224	0.156
	0.333	0.146	0.149	0.195	0.145
	-	-	0.164	0.109	0.072
	0.104	0.192	0.167	0.120	0.092
Semiaphis dauci	0.109	0.190	0.160	0.147	0.077
	0.094	0.210	0.171	0.125	0.085
	0.082	0.155	0.129	0.121	0.066
Cinara pini	0.293	0.142	0.153	0.230	0.189
	0.220	0.173	0.138	0.250	0.136

Lachnus pallipes	0.529	0.214	0.168	0.264	0.218
	0.469	0.138	0.113	0.243	0.185
	0.438	0.145	0.158	0.246	0.198
	0.535	0.312	0.185	0.312	0.265
	0.441	0.322	0.179	0.250	0.211
Aphis craccivora	0.234	0.148	0.261	0.147	0.092
	0.206	0.102	0.205	0.105	0.061
	-	0.082	0.146	0.067	0.067
	-	0.126	0.152	0.065	0.062
	0.252	-	0.136	0.082	0.066
Aphis fabae	0.323	0.145	0.235	0.141	0.114
	0.301	0.171	0.279	0.171	0.118
	0.259	0.124	0.219	0.163	0.091
	0.260	0.176	0.242	0.186	0.117
	0.259	0.138	0.233	0.190	0.100
Aphis hederae	0.269	0.128	0.175	0.103	0.063
	0.260	0.169	0.219	0.107	0.065
	0.212	0.110	0.174	0.122	0.054
	0.201	0.119	0.204	0.121	0.069
	0.200	0.102	0.192	0.122	0.082
Rhopalosiphum padi	0.129	0.180	0.123	0.109	0.056
	0.163	0.270	0.184	0.148	0.093
	0.179	0.307	0.184	0.148	0.093
	0.181	0.265	0.179	0.174	0.077
	-	0.266	0.164	0.152	0.078
Brachycaudus cardui	0.253	0.091	0.117	0.111	0.099
	0.255	0.126	0.096	0.131	0.119
	0.145	0.266	0.110	0.149	0.109
	0.154	0.275	0.103	0.175	0.100
	0.118	0.250	-	0.157	0.104
Dysaphis anthrisci	0.270	0.116	0.114	0.137	0.104
	0.263	0.104	0.125	0.126	0.103
	0.275	0.130	0.132	0.133	0.111
	0.258	0.079	0.108	0.139	0.091
	0.236	-	0.115	0.121	0.104
Dysaphis plantaginea	0.235	0.083	0.122	0.106	0.078
	0.223	0.109	0.123	0.135	0.077
	0.266	0.115	0.137	0.122	0.086
Dysaphis sorbi	0.276	0.088	0.118	0.139	0.099
	0.263	0.111	0.146	0.163	0.081

	0.218	0.102	0.115	0.132	0.089
	0.148	0.103	0.148	0.169	0.085
	0.108	0.279	0.125	0.147	0.098
Myzus cerasi	0.216	0.124	0.218	0.158	0.086
	0.238	0.128	0.208	0.159	0.075
	0.192	0.133	0.155	0.121	0.068
	0.282	0.128	0.229	0.143	0.098
	0.405	0.164	0.151	0.290	0.174
	0.359	0.166	0.134	0.283	0.174
Pterocomma rufipes	0.397	0.158	0.161	0.258	0.195
	0.489	0.143	0.177	0.303	0.252
	0.364	0.106	0.174	0.254	0.180
Thelaxes dryophila	0.183	0.080	0.114	0.131	0.066
	0.149	0.072	0.109	0.090	0.051
	0.188	0.065	0.122	0.110	0.056
	0.190	0.070	0.114	0.115	0.065
	0.179	0.080	0.125	0.096	0.064
