# Supplementary material for: Comparative Studies of Perianal Structures in Myrmecophilous Aphids (Hemiptera, Aphididae)
Source: Insects. 2022 Dec 16;13(12):1160. doi: 10.3390/insects13121160 (PMC9781728; doi:10.3390/insects13121160)
Supplement: Supplementary file 1 [file insects-13-01160-s001.zip › Supplementary Table S2.pdf]

Table S2: Mean values of measured structures [mm] (SD – standard deviation).

**species**	Anal plate length (mean/SD)	Anal plate width (mean/SD)	Cauda length (mean/SD)	Cauda width (mean/SD)	Cauda half width (mean/SD)
Glyphina betulae	0.241/ 0.039	0.064/ 0.009	0.147/0.021	0.061/ 0.009	0.116/ 0.021
Prociphilus bumeliae	0.262/0.009	0.392/0.005	0.093/ 0.013	0.216/0.033	0.173/ 0.013
Prociphilus fraxini	0.331/0.079	0.277/0.074	0.117/0.006	0.206/0.098	0.149/0.035
Symydobius oblongus	0.292/ 0.130	0.167/ 0.098	0.123/ 0.020	0.291/ 0.051	0.234/ 0.052
Panaphis juglandis	0.324/0.041	0.234/ 0.030	0.298/ 0.029	0.247/0.024	0.129/0.007
Chaitophorus nassonowi	0.259/ 0.037	0.109/0.022	0.103/ 0.008	0.143/0.015	0.075/ 0.015
Chaitophorus populeti	0.214/0.038	0.088/0.017	0.123/0.032	0.157/0.029	0.065/0.009
Aphis acetosae	0.131/0.013	0.216/0.020	0.195/0.043	0.137/0.027	0.217/0.304
Aphis jacobaeae	0.131/0.013	0.136/0.013	0.170/0.036	0.125/0.040	0.071/0.013
Aphis pomi	0.179/0.026	0.100/0.018	0.182/0.038	0.107/0.016	0.068/0.016
Aphis sedi	0.091/0.033	0.159/0.033	0.155/0.046	0.085/0.016	0.065/0.017
Brachycaudus tragopogonis	0.205/0.010	0.085/0.011	0.079/0.009	0.121/0.011	0.097/0.004
Anuraphis catonii	0.230/0.033	-	-	0.119/0.013	0.082/0.010
Metopeurum fuscoviride	0.234/0.065	0.215/0.084	0.2433/0.012	0.166/0.015	0.085/0.011
Pterocomma konoi	0.352/0.037	0.167/0.034	0.146/0.017	0.228/0.022	0.175/0.023
Semiaphis dauci	0.097/0.012	0.187/0.023	0.157/0.019	0.128/0.013	0.080/0.011
Cinara pini	0.256/0.052	0.157/0.022	0.145/0.011	0.240/0.014	0.162/0.037
Lachnus pallipes	0.482/0.047	0.226/0.088	0.161/0.029	0.263/0.028	0.215/0.030
Aphis craccivora	0.231/0.023	0.114/0.029	0.180/0.053	0.093/0.034	0.070/0.013

Aphis fabae	0.280/0.030	0.151/0.022	0.242/0.022	0.170/0.020	0.108/0.012
Aphis hederae	0.228/0.033	0.126/0.026	0.193/0.019	0.115/0.009	0.067/0.010
Rhopalosiphum padi	0.163/0.024	0.258/0.047	0.167/0.026	0.146/0.023	0.079/0.015
Brachycaudus cardui	0.185/0.064	0.202/0.086	0.106/0.009	0.145/0.025	0.106/0.008
Dysaphis anthrisci	0.269/0.006	0.117/0.013	0.124/0.009	0.132/0.006	0.106/0.004
Dysaphis plantaginea	0.244/0.018	0.096/0.018	0.121/0.011	0.125/0.013	0.087/0.011
Dysaphis sorbi	0.203/0.073	0.137/0.080	0.130/0.016	0.150/0.016	0.090/0.008
Myzus cerasi	0.232/0.038	0.128/0.004	0.202/0.033	0.145/0.018	0.082/0.013
Pterocomma rufipes	0.403/0.052	0.147/0.025	0.159/0.018	0.278/0.021	0.195/0.033
Thelaxes dryophila	0.178/0.017	0.073/0.006	0.117/0.006	0.108/0.016	0.060/0.007
