# Supplementary material for: Comparative Studies of Perianal Structures in Myrmecophilous Aphids (Hemiptera, Aphididae)
Source: Insects. 2022 Dec 16;13(12):1160. doi: 10.3390/insects13121160 (PMC9781728; doi:10.3390/insects13121160)
Supplement: Supplementary file 1 [file insects-13-01160-s001.zip › Supplementary Table S3.pdf]

Table S3: Mean ratios of measured structures

species	Anal plate length to cauda length	Anal plate lenght to anal plate width	Cauda length to half width cauda
Glyphina betulae	1.634	0.064	1.269
Prociphilus bumeliae	2.823	0.667	0.537
Prociphilus fraxini	2.836	1.195	0.783
Symydobius oblongus	2.363	1.743	0.526
Panaphis juglandis	1.089	1.386	2.314
Chaitophorus nassonowi	2.514	2.371	1.376
Chaitophorus populeti	1.734	2.434	1.906
Aphis acetosae	0.673	0.608	0.898
Aphis jacobaeae	0.769	0.966	2.407
Aphis pomi	0.979	1.790	2.690
Aphis sedi	0.587	0.573	2.383
Brachycaudus tragopogonis	2.606	2.398	0.812
Anuraphis catonii	-	-	-
Metopeurum fuscoviride	0.961	1.087	2.862
Pterocomma konoi	2.417	2.113	0.834
Semiaphis dauci	0.620	0.521	1.960
Cinara pini	1.763	1.629	0.895
Lachnus pallipes	3.004	2.133	0.746
Aphis craccivora	1.281	2.014	2.586
Aphis fabae	1.161	1.859	2.237
Aphis hederae	1.185	1.818	2.895
Rhopalosiphum padi	0.977	0.633	2.101
Brachycaudus cardui	1.737	0.918	1.003
Dysaphis anthrisci	2.177	2.309	1.167
Dysaphis plantaginea	2.013	2.524	1.388
Dysaphis sorbi	1.554	1.483	1.442
Myzus cerasi	1.146	1.809	2.477
Pterocomma rufipes	2.527	2.733	0.817
Thelaxes dryophila	1.522	2.422	1.934
